# The journey of discovery in co-creating knowledge to find a new way of working in municipal home care-seven lessons learned in a participatory appreciative action and reflection study

**DOI:** 10.1080/21642850.2025.2534624

**Published:** 2025-07-30

**Authors:** Inger James, Annica Kihlgren, Sofia Tavemark

**Affiliations:** a Faculty of Health, Science and Technology, Karlstad University, Karlstad, Sweden; b School of Health Sciences, Örebro University, Örebro, Sweden; c Örebro Municipality Healthcare and Social Services, Örebro, Sweden

**Keywords:** Fieldwork, co-creating knowledge, homecare, individual goal, participatory research, relationship

## Abstract

**Purpose:**

The study is part of a larger structural change programme, where Participatory Appreciative Action and Reflection (PAAR) has been used with the aim of changing home care practices to align with individuals’ needs and goals. The purpose of this study was to describe how the knowledge process in PAAR was conducted to develop a new way of working based on the individual’s needs and goals in home care.

**Method:**

A total of 160 co-researchers i.e. older persons, relatives, staff, administrators, first-line managers, case managers and persons from the authority were included in the study. Data was collected through fieldwork, including interviews, participant observations, informal conversations, focus group discussions, reference groups, and appreciative inquiry circles.

**Results:**

Co-creating knowledge was revealed as a three-step process: preparation for access to the field, being together in the field, and leaving the field. Each step describes several cycles of how the PAAR process proceeded, with actions leading to reflections and vice versa, which drove the knowledge process forward.

**Conclusions:**

The knowledge process of PAAR, gave rise to seven lessons learnt for future practice development: Contact pathways and trusting relationships, Loving struggle over time, An appreciative gaze, Patience and courage, Different ways of learning, A shared goal and Flexibility to adapt PAAR to changes in the field.

## Introduction

This study is part of a larger structural change programme aimed at transforming municipal elderly care to better address individual needs and goals. The focus of this study is to describe the methodologically-based knowledge process in applying interdisciplinary participatory research in practice and the lessons learnt. This research is based on the co-creation of practice in partnership with all parties involved. Participatory Appreciative Action and Reflection (PAAR) was used, the hallmark of which is to investigate and develop opportunities and success factors in practice, while seeking solutions to challenges (Ghaye et al., 2008).

Internationally, elderly care faces a major challenge in creating good living conditions for older persons. As the proportion of older persons with complex health problems and socio-economic difficulties is expected to increase (McNabney et al., 2022). At the same time, the health and social care workforce is shrinking (Figueroa et al., 2019; World Health Organization, 2022). Moreover, the World Health Organisation emphasises healthy ageing (World Health Organization, 2020) in partnership with the individual (World Health Organization, 2017; Patient act (SFS, 2024:821)). Older persons who need health and social care may prefer to receive home care (Chang et al., 2023; Government offices of Sweden, 2002; Tuckett A et al., 2018; World Health Organization, 2017), as an alternative to living in a nursing home. Because their home gives them a sense of belonging and identity (Gillsjö et al., 2011; James et al., 2019). However, home care and home health care have become increasingly complex (National Board of Health and Welfare, 2023; Shahriari et al., 2024). To meet the varied needs of older persons, structural changes are required, such as introducing more technological solutions in the home (Anttila et al., 2023; Landers et al., 2016; National Board of Health and Welfare, 2023), despite financial pressure on the organisation (James et al., 2024a; Shahriari et al., 2024). Older persons also experience insecurity, as staff may lack education (Bravell et al., 2021; Shahriari et al., 2024) and communication between older persons and staff may be poor due to the fragmentation of home health care (Bravell et al., 2021; National Board of Health and Welfare, 2023). For older persons, continuity (Hellzén et al., 2024; Tavemark et al., 2023), good quality home care, and the opportunity to express their needs and wishes are essential (Bravell et al., 2021; Dostálová et al., 2021; Tavemark et al., 2023). Reciprocity and respect between older persons and staff can generate positive and meaningful experiences (de São Jose et al., 2016; Hellzén et al., 2024; James et al., 2024b; Kihlgren et al., 2015; Olsen et al., 2022), where positive relationships with staff are crucial (Dostálová et al., 2021; James et al., 2024a; Jarling et al., 2018; Kihlgren et al., 2015; Olsen et al., 2022; Tavemark et al., 2023).

Elderly care worldwide is organised in different ways. In Sweden, elderly care is mainly municipalised, with elected local politicians holding the ultimate responsibility for the care of older persons (Hagerman et al., 2019; Gouverment offices of Sweden, 2017:2). Elderly care is divided into home care and home health care (Government offices of Sweden, 2002). Home care is regulated by the Social Services Act (Social Services Act (SFS, 2001:453)) and is carried out by care assistants and nurse assistants. Home care includes household chores, social support, personal hygiene, and grocery shopping (Tavemark et al., 2023). Home health care is governed by the Health Care Act (Health and Medical Care Act (SFS, 2017:30)) and the Patient Act (Patient act (SFS, 2024:821)). In home health care, nurses and occupational therapists are providing nursing, medical and rehabilitation care, and they also delegate these interventions to nurse assistants and care assistants (Tavemark et al., 2023). In this study, both home care and home health care are included and are hereafter referred to collectively as home care (HC).

Structural deficiencies in elderly care have been identified in Sweden, such as limited opportunities for older persons to influence the content of care and insufficient organisational resources (Andersson et al., 2022; Kihlgren et al., 2021; Ministry of Health and Social Affairs, 2010). However, efforts have been underway for a long time to address these shortcomings. As early as 2010, the Swedish government decided to introduce national guidelines for elderly care, which included core values and local dignity guarantees (Social Services Act (SFS, 2001:453); Ministry of Health and Social Affairs, 2010). However, staff describe it as difficult to apply core values as they experience organisational obstacles (Kihlgren et al., 2021). Furthermore, the Swedish Patient Act, introduced in 2014, aimed to shift power to the patient, with decisions being made in consultation between staff and the individual (Patient act (SFS, 2024:821)). However, an evaluation described the Patient Act as ineffective, as staff do not follow what the act stipulates (The Swedish Agency for Health and Care Services Analysis, 2017). There is a strong drive for HC to invite older persons to participate and cooperate (Lidström-Holmqvist & James, 2019; State public reports, 2022:41; Ministry of Health and Social Affairs & SALAR, 2023; Tavemark et al., 2023) and for care to be based on individuals’ needs and goals (Bravell et al., 2021; National Board of Health and Welfare, 2021). Thus, structural changes are underway within HC (Lidström-Holmqvist & James, 2019) to shift power and move from a top-down to a bottom-up perspective, where older persons and employees are at the centre (National Board of Health and Welfare, 2021) and can influence the design of working methods in HC (Tavemark et al., 2023). This aligns with the Swedish government’s trust reform, which aims for the public sector to have governance based on trust and confidence in the experience and knowledge of employees (Government Public Inquiries, 2019). Research that demonstrates feasible pathways and constructive evaluations of the implementation of new working methods has been requested by the authorities (The Swedish Agency for Health and Care Services Analysis, 2024). PAAR can then be used as a strategy to develop ways of working (Tavemark et al., 2023), where the participants create a dynamic interaction and equality between research, learning, and change, in partnership with the researchers. Through this, participants can improve their own practice together (Ghaye et al., 2008; James et al., 2015; Magnussen, 2019; Reason & Bradbury, 2008) and develop a new way of working (reported elsewhere). To learn how knowledge can be co-created in practice to change and develop ways of working, it is important to describe what happens during the methodological-based knowledge process in PAAR.

The purpose of this study was to describe how the knowledge process in PAAR was conducted to develop a new way of working based on the individual’s needs and goals in home care.

## Method

### Methodological and theoretical approaches

The participatory research method involves engaging individuals who have experience with the problem in a knowledge process, viewing them as co-researchers. The researchers participate as research facilitators, guiding discussions and contributing current research and theory where appropriate, to learn together through pluralism and reflection (Ghaye et al., 2008; Melander-Wikman et al., 2008; Reason & Bradbury, 2008). Central to PAAR is the assumption that knowledge is gained by creating symmetry and robust relationships (Ghaye, 2010; Ghaye et al., 2008). To create interactions in the construction of knowledge (Ghaye, 2010), research facilitators must have social skills and be able to form connections with people (Ghaye et al., 2008). Research facilitators, need to apply an appreciative gaze and show positive engagement, appreciating co-researchers’ experience and knowledge to create an appreciative space (Ghaye et al., 2008; Melander-Wikman et al., 2008). Human flourishing could include what an individual, a team, or an organisation can consider as their capabilities, supporting democratic values and a common goal (Ghaye, 2010).

**Figure 1.**
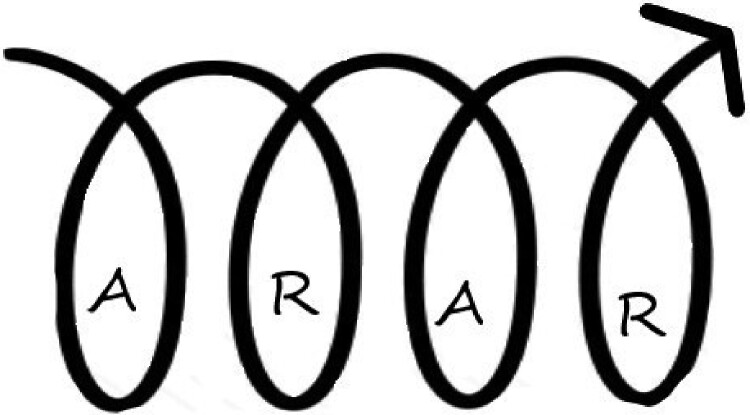
Illustration of the cycles describing how action and reflection advance the knowledge process.

The participatory research process changes according to its context and what is happening in the field (Arnold, 2020; James et al., 2015; Reason & Bradbury, 2008). PAAR integrates action and reflection in an interdependent relationship, where action shapes reflection and reflection shapes action (Ghaye, 2010; Ghaye et al., 2008; Reason & Bradbury, 2008). It is a cyclical process that incorporates feedback on results based on collected data and reflection (Petersson & Rämgård, 2023). Reflections lead to insights that drive the project forward in action (see Figure 1) and guide decisions made to further the process (Arnold, 2020), while practical implementation should take place at the same time. The use of research circles, in the form of appreciative inquiry circles (Cooperrider et al., 2008), in participatory research (Havens et al., 2006; Holmstrand et al., 2008; Holmstrand & Härnsten, 2022; Magnussen et al., 2019) is effective in challenging structures and establishing new practices (Ghaye, 2010; Havens et al., 2006), for a sustainable implementation of a new way of working. The appreciative inquiry circles operated as workshops with staff and research facilitators, building on Cooperrider’s cyclic 4-D phases: discover, dream, design, and destiny (Havens et al., 2006) where each phase can be adapted to the field and participants during the process.

### Selection process

All home care units in a municipality, (n = 20) were supposed to participate, the top management selected three home care units to start with, depending on which units had capability for participating. To get variation and learn from the participants, a strategic selection process was used in the first step, which included three new HC units: a central city HC unit, a suburban HC unit, and a rural HC unit. Three more HC units were asked to participate during the process, one of which declined. Research facilitators (referred to as RFs) used a convenience sample of co-researchers (Polit & Beck, 2017 [2018]) (see Table 1). ‘Co-researchers’ refers to all project participants who were asked to participate i.e. older persons, relatives, staff, administrators, first-line managers, case managers and persons from the authority. Older persons who were 65 years and older in ordinary housing with HC were asked to participate. If they consented, a designated relative was also asked to participate. Staff from the included HC units, were also asked to participate; nurse assistants (care assistants and nurse assistants referred to collectively as nurse assistants), occupational therapists and registered nurses are all referred to collectively as staff. The RFs consisted of a research assistant (ST) and a researcher (IJ).

**Table 1.** Description of co-researchers.

	Total (n):
Older persons (individuals 65 years and older)	71
Relatives to Older person	8
Nurse assistants	40
Occupational therapists	8
Registered nurses	11
First line managers and administrative staff	17
Case manager	3
Persons from authority	2
A total of 160 co-researchers were included in this study.	

### Data collection and analysis

In total, 160 co-researchers gave written informed consent to participate in the study. Co-researchers could choose to participate in several or some parts of the data collection, which included interviews, participant observations, informal conversations, focus group discussions, reference groups, and appreciative inquiry circles (see Table 2). The interviews, focus group discussions and reference groups were audio recorded. Participant observations, informal conversations, appreciative inquiry circles were audio recorded and, in addition, field notes were made. All data were transcribed verbatim by a professional transcriber. The description of the knowledge process in this study is based on diary entries for reflection and tracking of the knowledge process.

**Table 2.** An overview of the individual interviews, participant observations with informal conversations and fieldnotes, Focus Group Discussions (FGD), Reference groups (Ref-gr) and Appreciative inquiry circles (AI-circle). Due to Covid-19, some interviews and FGD were held via telephone/digital video meetings replacing physical individual interviews (marked with *). The co-researchers could participate in one or two interviews, FGD or Ref-gr, they were asked for participation continuously during the process. There were 45 older persons who only participated in participant observations when the RFs followed the professionals.

Diary was written continuously, approximately 70 pages, Times new roman, font size 12 with 1,0 line spacing																	
	Individual interviews	Second individual interviews	Participant observations with informal conversations and fieldnotes ()= time for recording and writing fieldnotes	FGD unit 1	FGD unit 2	FGD unit 3	FGD unit 4*	FGD unit 5*	FGD unit 4, 5*	FGD unit 4, 5*	FGD*	Ref-gr unit 1	Ref-gr unit 2	Ref-gr unit 3	AI-circle unit 1	AI – circle unit 2	AI circle unit 3
Older persons (≤ 65 years)	26 (*3)	4										2	3	1			
Relatives	6 (*1)	0										2					
Nursing assistants	21	0	15	4	2	4	6	5				4	4	20	4	3	25
Occupational therapists	8	1	6	3	2	1						4	2	2	3	2	1
Registered nurses	8	0	5	1	1	2						2	1	3	1	3	4
First line managers	8	3		2	1	1				2		3	2	1	2	1	1
Administrative staff	6	1	1	1		1			3			1	1	2	1	1	2
Case manager											3				3		
Persons from authority						1							1	2			1
**Total number of co-researchers (n)**	83	9	27	11	6	10	3	5	3	2	6	15	14	34	14	10	34
**Total hours per method**	83 × 1,5 h	9 × 0,5-1,5 h	27 á 5 h (27 á 4 h)	1,5h	1,5h	1,5h	1,5h	1,5h	1,5h	1,5h	1,5h	1,5h	1,5h	1,5h	2 × 1,5h	5 × 1,5 h	4 × 1,5 h
**Total hours of fieldwork**	**410 h**																

According to De Oliviera (De Oliveira, 2023) participatory action research can be problematic for facilitators to apply due to power relations between co-researchers. During data collection, the RFs were aware of and managed power relations to create good relationships between the different groups of co-researchers and between co-researchers and RFs, to create symmetry and maintain a bottom-up perspective to enable co-creation of knowledge. The RFs and co-researchers had different roles during the different steps of data collection and the analysis. In order to equalise the power relations, the RFs had the role of facilitator and started all meetings by informing about the project, the common goal and that the co-researchers and RFs were there to learn from each other. Throughout the data collection processes the RFs have been open and they used reflective questions in an everyday conversation to learn about the co-researchers’ reasoning on how a new way of working could be developed. In their role as facilitators the RFs also integrated research and theory to enhance their reasoning and help create knowledge together.

The older persons’ and the relatives’ roles became one of co-creating knowledge. They shared their wishes and ideas about what needed to change in HC. In the reference group, they presented their ideas for a new way of working directly to the invited people in leading positions and actively participated in discussions based on their experience and knowledge. Staff, administrators and first-line managers were involved in all aspects of data collection, but their roles could vary. In interviews, participant observations and informal conversations they shared their thoughts and ideas on how HC needs to change. In the focus group discussions and reference groups, they discussed the wishes of the individual and actively drove the discussions. They also expressed their thoughts directly to people in leadership positions and supported the perspectives of older persons in the reference groups. Staff ran the appreciative inquiry circles and documented the work as they tested new ways of working.

During data collection, the RFs were aware of the power relations and therefore divided the reference groups into smaller groups. This was mainly because older persons and their relatives can be in a vulnerable position. In addition, the RFs were ready to support by guiding and creating space for older persons and relatives to express their perspectives. Nurse assistants can also be in a vulnerable position in relation to their first line manager. Therefore, the RFs encouraged and empowered them to share their knowledge in the focus groups discussions, where the first line manager also participated. First line managers, administrators, occupational therapists and registered nurses can also be in a vulnerable position as they have a managerial role and may be held accountable for the shortcomings in HC. The RFs therefore provided guidance on the meaning of PAAR, that is to focus on opportunities, creative solutions and suggestions to reduce obstacles, rather than looking for who is responsible for the shortcomings.

**Figure 2.**
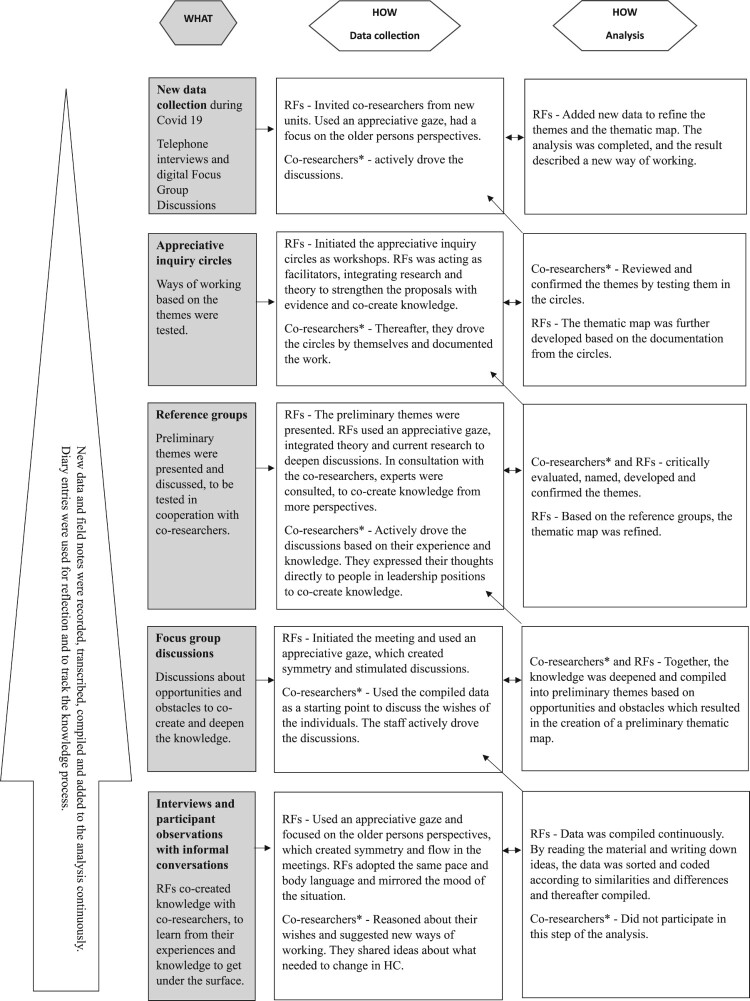
A description of what was done (grey) and how (white) during the co-creation of knowledge and how the analysis inspired by Braun and Clarke's thematic analysis was conducted. * Co-researchers refer to those who participated in the various data collections.

Data collection and analysis were carried out simultaneously in a fruitful co-creation of knowledge (see Figure 2). All data were combined and treated as a whole at all stages of the analysis, inspired by Braun and Clarke's thematic analysis (Braun & Clarke, 2006). Data was analysed continuously with each new set of data added. Data collection led to analysis, which led to new data collection, which in turn became a means of processing and confirming new data and continuing the analysis. In the analysis, the role of the RFs was to first read the material, and write down ideas, followed by sorting and coding the data for similarities and differences. The data was then compiled. Together in focus group discussions, co-researchers and RFs had the role of further analysing the compiled data and creating preliminary themes based on opportunities and obstacles. Relationships between different levels of themes were analysed, resulting in a preliminary thematic map.

In the reference groups, co-researchers and RFs worked on critically evaluating themes and testing different names to develop and confirm themes. The thematic map was refined by the RFs. In the appreciative inquiry circles with staff, administrators and first line managers, new ways of working based on the themes were tested. Depending on the feedback from the tests carried out by staff, administrators’ and first line managers, the themes could be adjusted and confirmed. According to the thematic analysis, the RFs developed the thematic map based on the documentation of the circles of appreciative inquiry. As new data were collected, the RFs added data to the analysis to develop and finally refine the themes and thematic map. The RFs compared the themes in relation to labels and content in order to increase trustworthiness. Finally, a new way of working was described (reported elsewhere).

### Ethical considerations

Prior to the start of the project, several ethical considerations were carefully addressed, and an ethics application was submitted to and approved by the Swedish ethical review authority (Dnr: 2019-02575). To learn from older persons with diverse needs, knowledge, and experiences, a decision was made not to exclude persons with complex care needs and cognitive impairments. While providing informed consent to participate is more difficult when including such persons in research, to exclude them altogether may also be considered unethical because of the principle of unfair discrimination and exclusion from benefits of participation (Szala–Meneok, 2008). We asked the first line managers to inform and ask older persons who were potential participants in a way that was suited to the individual, to reduce the risk of harm, e.g. for those who were suffering from mental illness or anxiety (Tavemark et al., 2023). Potential participants were also given verbal and written information and provided written informed consent if they agreed to participate (James et al., 2015). Furthermore, information about the research was disseminated in every meeting. The RFs paid attention to the co-researchers’ body language and how they spoke to make sure that they did not display reluctance to participate (Tavemark et al., 2023). They were also informed that they could end their participation at any time without giving an explanation (James et al., 2024a).

## Results

### Results of co-creating knowledge in the field

The results are presented as knowledge co-created in the field based on three steps: preparation for access to the field, being together in the field, and leaving the field. Each step includes several cycles that describe how the PAAR process, where actions (A) led to reflections (R) and vice versa, driving the knowledge process forward (see Figure 1).

#### Step 1: preparing for access to the field

*The first cycle-Start up and establishing cooperation*

**A** When HC in the municipality initiated the structural change programme, an information meeting was held for all staff. The researcher IJ was invited to speak about earlier research from previous projects about structural changes in the organisation.

**R** A senior manager and IJ reflected that structural change ought to be carried out together in line with participatory research, which should be anchored in those it concerns.

**A** As a result, HC management decided to implement the structural change programme ‘working according to the needs and goals of the individual’ using a participatory research approach. To start the project, a research plan and an ethics request had to be designed and submitted to the Swedish ethical review authority. The research plan stated that the RFs would follow the process of co-creating knowledge together with the HC units conducting the data collection while keeping diaries of reflective notes.

**R** The diary entries aimed to enable reflection and tracking of the knowledge process in PAAR over time, to facilitate understanding of what was being co-created in the field and what might need to be changed during the process.

**A** When the ethics application was approved, a steering group was formed with the mandate to make decisions and facilitate cooperation between the municipality and the university. The steering group included two senior managers responsible for the cooperation with the local politicians for elderly care and a university professor (AK) who was responsible for the research project. The senior managers appointed a coordinator who was part of the management team of the structural change programme.

**R** The RF team was perceived as well suited, with ST, with a MSc and background as an occupational therapist within HC, providing insight and contacts that could create access to the field, and IJ, with a PhD, providing theoretical knowledge of participatory research and experience with previous research projects in the municipality. The involvement of the coordinator was considered important, as she/he had contacts and knowledge of the organisation through her/his management role, which could facilitate access to the field in HC. Overall, the steering group, the RFs, and the coordinator were key persons in gaining access to the field and establishing cooperation.

#### Step 2: being together in the field

*The second cycle-Seeking access to the field*

**A** The steering group selected the three HC units, and first line managers were informed about data collection and given the opportunity to ask questions during a meeting with the RFs. Subsequently, staff in the HC units were informed and those who agreed to participate were included. The administrator of each unit provided access to the field. For example, administrators would schedule the nurse assistants to open the door for RFs when they arrived at the homes. When interviews were over, RFs called the administrators to let them know that the staff could now lock the door. Some older persons consented to the interview and the reference group, while others consented only to the researchers entering the home to observe the staff at work. At the end of each interview, the older person was asked if they allowed the RF to contact their relatives for an interview.

**R** However, the diary entries revealed that RFs experienced obstacles to accessing the field because first line managers were busy, and it took time to reschedule appointments. All first line managers expressed concern that having staff involved would take time away from their daily work. The RFs realised that additional information about the project was needed to try to ease access to the field.

**A** The coordinator therefore attended further information meetings to reassure first line managers that the decision to support the project had been made at senior management level and to encourage participation. Furthermore, they clarified that temporary workers could replace regular nurse assistants in their ordinary work to allow them to participate as co-researchers.

**R** The coordinator’s participation improved access to the field in this early phase of the project.

*The third cycle-Striving for a bottom-up perspective*

**A** RFs started data collection with interviews and carried out participant observations with informal conversations in the field, but staff participation remained low.

**R** The RFs reflected on the hesitation of staff to participate and attributed their uncertainty possibly to a fear that senior managers would find out what they had said.

Furthermore, the staff were reluctant to express their ideas as it could go against HC, which they felt had a focus on finances, i.e. changes must not burden the budget. RFs experienced a struggle with power relations and assisted staff to develop enough confidence to talk and think freely, to maintain the bottom-up perspective.

**A** Another meeting with the first-line managers was attended by a representative from the steering group and first-line managers were informed that they did not have access to the data. The first-line managers then ensured the staff that the steering group did not have access to any data.

**R** The RFs reflected with the coordinator that it was difficult to maintain a bottom-up perspective. The coordinator then expressed a view that the organisation was working with consultants on a change of management in HC, using buzzwords such as ‘efficiency’ and ‘balanced budget.’ A competition emerged in the field between the consultants’ use of buzzwords and the research’s focus on the needs and goals of individuals with HC. This led to a lack of consensus and disagreements between the coordinator and the RFs about the research needing to be grounded in the co-researchers’ perspective.

**A** Due to the lack of consensus, another meeting was held where the steering group reinforced the difference between organisational development and research, clarifying the role of the coordinator to stimulate research and maintain the bottom-up perspective. The meeting also emphasised the importance of not initiating additional changes in the HC units involved in the project.

**R** This meeting with the steering group, grounded in trust, resulted in improved cooperation with the coordinator, who took on a more supportive role and communicated more effectively about the project. This led to increased interest in participation among the staff. The RFs realised that as the number of participants increased, more RFs were needed from the university to conduct data collection.

**A** Two persons agreed to join the project as RFs. To reach consensus, the original RFs held information sessions about the democratic approach, emphasising the significance of using an appreciative gaze, being humble, and showing genuine interest.

**R** While the original RFs analysed the conduct of the new RFs’ interviews, they reflected and concluded that the interviews were too structured and rigid. For example, there was a focus on the older persons’ routines without hearing their voices, there was a lack of goal descriptions and ideas for changes in HC. This led to a top-down perspective where symmetry in the co-creation of knowledge was lacking. The struggle was described in the diary entries, and we learned the importance of RFs having experience in both participatory research and HC to deepen conversations in the interviews.

**A** Several meetings were held among the RFs to reach a shared understanding of participatory research, but consensus was not achieved. This resulted in the original RFs continuing the data collection alone. At the same time, the coordinator started questioning the necessity of including so many co-researchers, due to the financial burden on HC.

**R** The RFs perceived the discussion as a continuing struggle caused by a lack of consensus. RFs were thinking about the older persons and PAAR, while the coordinator was thinking about organisational development, emphasising efficiency and a balanced budget in HC. Conflict arose due to the lack of consensus.

*The fourth cycle-Flourishing in the co-creation of knowledge*

**A** The conflict led to a meeting with discussions about whether the voices of older persons were being heard and if the focus was on their perspectives. The meeting ended in a consensus and the coordinator suggested that the term ‘goals’ could be replaced by ‘wishes’, as it is more in line with how persons commonly express themselves.

**R** The RFs experienced interview conversations flourishing when the co-researchers talked about ‘wishes’ and reflected on the fact ‘goals’ were perceived as abstract and demanding. It was also because older persons said that major life goals disappeared as they got older. It was easier to talk about wishes, and the difficulty of distinguishing between needs and wishes was described.

**A** Reflective questions were rephrased, with ‘wishes’ used instead of ‘needs’ and ‘goals’ in interviews that were held as everyday conversations. For example, topics included what was good or bad about HC and its impact on daily life. Furthermore, what needed to change in HC to meet individuals’ wishes was discussed. All co-researchers were offered a second interview, allowing RFs to get further under the surface of co-creation and to get explanations. An appreciative gaze was used, which created symmetry and flow in the meetings. The longer the RFs were in the field and recognised by the co-researchers, the more potential participants became interested and involved and wanted to participate as co-researchers. The RFs decided to stay in the field and work with more co-researchers.

**R** On the one hand, it became a challenge to keep up with all the data collection but on the other hand, as RFs and co-researchers co-created knowledge, trust and cooperation developed, resulting in more data for co-creating knowledge. Co-researchers dared to open up and suggest new ways of working.

**A** At the same time as the interviews were taking place, RFs were ‘shadowing’ the staff in their daily work. RFs observed what they did and adopted the same pace and body language to reflect the mood of the situation. RFs wore HC uniforms to blend in and helped with simple tasks such as passing items, taking out the garbage, or opening doors. Informal conversations were held during or shortly after the home visits, when RFs and the staff reflected and learnt from explanations of what happened in various situations. They also reflected on why things were done in a special way and how a person’s wishes could be met. For example, one nurse assistant was observed reheating food in batches and adding fresh vegetables to the finished dish. In an informal conversation the RF was able to receive the following explanation: different foods need to be reheated in different batches to taste good. The nurse assistants also knew that the individual appreciated vegetables.

During the observations, with the older person’s consent, RFs noted that more older persons and their relatives wanted to share their everyday experiences through interviews.

**R** Contradictions emerged during the interviews and participant observations with informal conversations. For example, in the interviews the staff emphasised the importance of sitting down with older persons to meet their wishes. However, in the participant observations, RFs were able to study the body language of the co-researchers and saw them talk to older persons while engaged in something else. Through informal conversations, it was revealed that this contradiction was caused by a lack of time.

**A** During the car and bicycle rides to and from the older persons’ homes, informal conversations were held with the staff, where they shared ideas about what needed to change in HC for it to better meet individuals’ wishes. In addition to making field notes, RFs also documented their experiences of the fieldwork in the diary, and these notes were used during the knowledge development process.

**R** Diary entries revealed feelings of danger when RFs navigated busy roads on their bicycles. The RFs were also afraid of delaying nurse assistants if they took too much time putting on their jacket or unlocking their bike. This allowed RFs to ask how time pressure affected their ability to meet individuals’ wishes.

**A** Data from interviews and participant observations with informal conversations were transcribed and analysed inspired by Braun and Clarke's thematic analysis (Braun & Clarke, 2006) (see Figure 2). By reading the material and writing down ideas, the data was sorted and coded according to similarities and differences and thereafter compiled. The focus group discussions were then initiated, where the staff, administrators and first line managers used the compiled data as a starting point to discuss the wishes of the individuals. Together, the knowledge was deepened and compiled into preliminary themes based on opportunities and obstacles, which resulted in the creation of a preliminary thematic map.

**R** In the focus group discussions, the staff, administrators and first line managers who had different backgrounds and levels of experience, contributed to deeper discussions and a wider range of perspectives on how HC could better align with individuals’ wishes. RFs were aware of power relations and used an appreciative gaze, which created symmetry and stimulated discussions. The staff actively drove the discussions facilitating the co-creation of knowledge and fostering trust thereby enhancing understanding of how opportunities and obstacles impacted HC.

**A** The preliminary themes were then taken back to the co-researchers, who were invited to participate in reference groups. Older persons were provided with transport to the HC unit to make it possible for them to participate. The preliminary themes were presented in paper form for the co-researchers to critically evaluate develop, name and confirm them based on the discussions. In the reference group, the RFs used an appreciative gaze, integrating theory and current research to deepen discussions. The importance of relationships for person-centred HC was described, and when the meaning of goals was discussed, one older person commented: ‘the wish can be just to have some fun.’ A good relationship is the key to knowing the older person’s wishes. This was confirmed by other co-researchers, which shifted the process of building knowledge, causing the preliminary themes to ‘move around.’ In consultation with the co-researchers, experts with specific knowledge and positions of authority in HC, such as case managers, were invited, as co-researchers, to co-create knowledge from more perspectives. Based on the reference groups, the thematic map was developed, confirmed and refined. Interest in participation remained high, and several reference groups were held per HC unit (see Table 2).

**R** The discussions in the reference groups were fruitful and flourished, with the co-researchers actively driving the discussions based on their experience and knowledge, which developed the preliminary themes. There was symmetry in the discussions, with older persons and relatives appreciating being able to express their thoughts directly to people in leadership positions. They described opportunities and obstacles in working according to individuals’ wishes and suggested that HC should be adapted to individuals’ routines, for example, the importance of replacing cleaning with a coffee break was identified as an opportunity. An obstacle to individuals’ wishes could be, for example, if an older person wanted to sit up and finish watching a football match when an NA was scheduled to provide bedtime support.

**A** As a step in implementing a new way of working, the refined themes were brought back to the staff, administrators and first-line managers. Together we conducted appreciative inquiry circles to test new ways of working based on the themes. The RFs initiated the first appreciative inquiry circles in the form of workshops. Furthermore, the RFs acting as facilitator, integrating research and theory to strengthen the proposals with evidence and co-create knowledge. Thereafter, staff drove the circles by themselves and documented the work. The staff, administrators and first line managers began by highlighting things they were doing well in order to *discover.* They then talked about how they followed the individuals’ wishes, *their vision*. Thereafter, they worked on how to make their vision *a reality*, i.e. how to develop interventions that work according to individuals’ wishes. The final step was to work with implementation of the intervention, *to preserve* (Cooperrider et al., 2008)*.* The groups worked with several proposals, with one group wanting to improve the introduction of new colleagues, to ensure that they had the knowledge they needed to make everyday life work for the older person. Depending on the feedback from the tests carried out by staff, administrators’ and first line managers, the themes could be adjusted and confirmed.

**R** Some staff expressed a view that first-line managers were important in the appreciative inquiry circles to make suggestions and decide which proposals they could start to implement in HC. RFs considered that, while this could mean a top-down perspective if staff did not dare to express themselves, it could also be a way to promote working together, regardless of position, to implement a new way of working.

**A** Insights from the appreciative inquiry circles were added to the themes, and the thematic map was further developed. It was time to report back to the steering group and local politicians based on the progress seen in the first three HC units and start planning the next steps of the project. However, this cycle of the project coincided with Covid-19 escalating in society, and the pandemic being declared.

*The fifth cycle-Lockdown and the field closed*

**R** The spread of Covid-19 interrupted the previously thriving project, shifting HC’s focus and changing working methods. The staff only conducted the most necessary home visits to meet the health needs of older persons. The restrictions also limited the RFs’ ability to be in the field. Whether and how the project could continue was uncertain.

**A** After feedback, the steering group decided that three new HC units, of which two accepted, could be included, and the RFs switched to digital data collection via zoom instead of physical meetings. Extensive planning and ethical considerations were required to adapt the project to the terms of the pandemic, and an updated ethics application was prepared.

Due to the pandemic restrictions, participatory observations, informal conversations, reference groups, and appreciative inquiry circles could not be carried out. The staff, administrators and first line managers took actively part in digital focus group discussions which they drove, instead of individual interviews. This was to speed up data collection, as we did not know how long we would be able to co-create knowledge in the field. Older persons and their relatives who wanted to participate in individual interviews were offered telephone interviews. In these, the RFs used an appreciative gaze and focused on the older persons perspectives. The new data collection (see Table 2) contributed to developing and refining the themes and the thematic map (see Figure 2).

**R** The telephone interviews with older persons and relatives provided surprisingly rich insights and focused on the person, possibly due to the questions being asked more openly and there being nothing else to distract the RFs. For example, interviews were not guided by what the RFs could see in the home, such as assistive devices and medical supplies. Instead, the persons’ story was the focus of the interviews.

However, it became clear that it was difficult to make room for research in HC due to the pandemic straining resources. More staff called in sick, and mortality increased among older persons.

**A** Based on the situation in HC, the steering group reconsidered the duration and impact of the project on the daily lives of older persons and staff during the pandemic, leading to the decision to stop the project. RFs completed the thematic analysis using Braun and Clarke’s method and described a new way of working.

**R** The co-researchers expressed their disappointment when the RFs left the field. Because the RFs had been interested and valued them as co-researchers.

**Figure 3.**
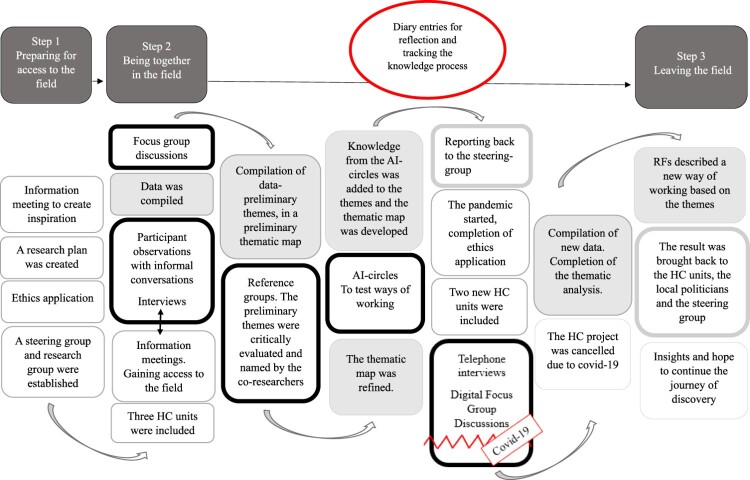
Illustrates the PAAR process with the co-researchers and the interruption of the pandemic. Co-creating knowledge in the field. The different colours symbolise different parts of the process. Dark grey – Steps in the knowledge process. Red frame – Reflection and keeping track of during the process. White – Establishing and ending cooperation. Black frame – Data collection and analysis together with co-researchers. Grey – RF´s compilation of data. Grey frame – Bringing the result back to HC.

Despite the interruption, a large amount of data was collected, describing how the knowledge process was carried out in PAAR to develop a new way of working based on the wishes of the individual (see Figure 3).

#### Step 3: leaving the field

*The sixth cycle-Insights and hope*

**A** The new way of working was presented to HC units, the steering group, and local politicians. First-line managers received a summary of the result so far.

**R** Reflecting on the project, it was possible to conduct research under difficult circumstances and produce results despite reaching only five HC units of the 20 HC units it had been planned for. In retrospect, the data was extensive and could have been the basis for a preliminary result earlier in the knowledge process. Focus group discussions, reference groups, and appreciative inquiry circles could also have been initiated earlier in new HC units to refine the knowledge and test a new way of working. The staff questioned why older persons and relatives were not included in the focus group discussions and appreciative inquiry circles. We realised that they were not involved in developing and testing the new way of working, meaning that the main persons’ voices were not heard to the extent intended.

The project ended in a pandemic when no one knew what the future held. The hope is to be able to access HC again to further develop the thematic map in reference groups and conduct appreciative inquiry circles. The vision is to involve all HC units with older persons and relatives to implement a new way of working. Hopefully, the knowledge process will continue in a new project.

## Discussion

### Tensions between struggle and flow in PAAR

The study described the knowledge process in PAAR to develop a new way of working in HC based on individuals’ wishes, highlighting what opportunities there were and what obstacles appeared along the way. Participatory research can be seen as a journey of discovery and co-learning to develop practice together. Therefore, it became important for us to keep diary notes of the content and reflections of the knowledge process (Lindhult, 2022). The notes also showed that we were in a tension between struggle and flow, which created flourishing during the process, and led to actions and reflections driving the knowledge process forward. Struggles could at times be perceived as conflicts when the participants have different goals, but according to Jaspers (Wallraff, 1970), there is no conflict if there is a shared goal, which can then be defined as a ‘loving struggle.’ In this project, the shared goal was to work for the individuals’ wishes. Through reflection, we were also able to distance ourselves from our own preconceptions and understand how others saw the situation (Ricœur, 1993). This provided a flow that can be described as a psychological state in which people are engaged in pleasurable and challenging activities (Csíkszentmihályi, 1996). In the discussion, we have chosen to use the concept of flow according to Csíkszentmihályi (Csíkszentmihályi, 1996) and the concept of loving struggle according to Jaspers (Walraff, 1970) to illustrate the tension in the knowledge process.

#### Contact pathways and trusting relationships as bridges to the field provide flow

The first lesson we learned on the journey of discovery was the importance of having contact pathways to build relationships and trust in HC, which provided flow. Contacts from previous HC projects enabled project initiation (James et al., 2015; James et al., 2019). The formation of the steering group was also crucial to get the mandate to conduct the project and to gain access to the field. The steering group and RFs together with the coordinator can be seen as a platform, consisting of a small number of persons with a common dedicated commitment that built bridges between people (James et al., 2015; Lindhult, 2022), providing a flow in the knowledge process.

RFs enriched and learned from each other as they came from different organisations with different experiences and professions. ST had knowledge and contacts in HC, which could have led to an insider perspective, i.e. that one’s own activities and practices are studied (Coghlan, 2014). The researcher IJ had a theoretical knowledge of PAAR and an outside perspective on the practice being studied. The different positions of insider and outsider, combined in mutual partnership, resulted in what can be called the most democratic approach (Andersson, 2022; Herr & Anderson, 2005). The shared knowledge process of the RFs created symmetry and gave us trust in each other. Our trust also led to trust in co-researchers, which created relationships and bridges into the field.

#### Loving struggle over time is important to strengthen relationships

The second lesson we learned on the journey was the loving struggle of needing time to build contacts and relationships. Despite good contacts and key persons, it was a struggle to get access to the field. Information meetings with first-line managers often had to be rescheduled due to lack of time. Other studies have also found that the maturation process of making contacts and forging relationships takes time (Bergdahl, 2022; Magnussen, 2019), but when it succeeds, motivation and participation follow (Dewar & Kennedy, 2016; Magnussen et al., 2019).

During the struggle, we also discovered that guarantees and conditions from the senior manager in the HC are crucial, as they support and encourage participation (Reason & Bradbury, 2008). This shows that participatory research needs to be continuously anchored at several levels from senior management to co-researchers (Bergdahl, 2022; Reason & Bradbury, 2008) allowing the knowledge process to move forward.

#### Using an appreciative gaze provides flow and a bottom-up perspective for practice change

The third lesson we learned was that flow was achieved when we used an appreciative gaze, which is crucial for co-researchers’ engagement in knowledge development and practice change (Ghaye & Lillyman, 2008; James et al., 2015; Magnussen et al., 2016). RFs showed appreciation for co-researchers’ experiences and knowledge and used attachment for bonding, which provided symmetry and a bottom-up perspective (Ghaye, 2010). The importance of an appreciative gaze and humility were seen to be essential to creating interactions with potential co-researchers. After some time, more participants wanted to join the project as co-researchers, which created further flow and allowed us to stay longer in the field. In PAAR, an appreciative gaze should be used to show genuine appreciation and kindness as it can enhance co-researchers’ feelings of joy and satisfaction in their work (Ghaye, 2010). This became evident when the project ended and left co-researchers feeling empty with several wondering: ‘Who will be interested in our work now’?

#### Patience and courage are crucial in loving struggle to keep the bottom-up perspective

The fourth lesson and perhaps the most crucial we learned was the importance of having the patience and courage to have a loving struggle to keep the bottom-up perspective. Different interpretations of PAAR and what collaboration with co-researchers meant emerged throughout the project. This may have been because we did not provide enough information to the new RFs, which could be seen as a weakness in the process. On the other hand, it can be difficult to clarify and explain PAAR to outsiders who do not have previous experience with participatory research (Canlas & Karpudewan, 2020). However, it may ultimately be about the ontological perspective i.e. which scientific paradigm is preferred (Polit & Beck, 2017 [2018]). If we think that there is a truth, an ordered reality, where the world can be represented with measurable objective evidence, i.e. positivism (Polit & Beck, 2017 [2018]), it becomes impossible to conduct PAAR. A hermeneutic perspective, which centres on a person’s lived experience, is needed here, i.e. how the individual experiences and understands his/her world (Polit & Beck, 2017 [2018]). It is about learning from participants and creating a partnership, which requires the researcher to renounce the privileged position of ‘expert’ and adopt a solidarity-based co-researcher stance that recognises that people have valuable knowledge and insights. Building partnerships, not extracting data, should be the guiding principle (Salzmann-Erikson, 2024). Participatory research is not a method but an approach with a democratic orientation (Lindhult, 2022).

The loving struggle to maintain the bottom-up perspective in the process may also have been further complicated by the presence of HC consultants who worked according to other values such as cost-effectiveness i.e. a budget in balance. This may have created a polarisation in the field between the consultants’ rhetoric and the research focus on the individuals’ wishes with their HC. It is difficult to conduct PAAR in an organisation governed by the principle of cost-effectiveness (James et al., 2024a; Tavemark et al., 2023). We had a loving struggle with the coordinator, who partially represented HC’s economic perspective, where discussions with each other about the meaning of PAAR became a strength. We learned that questions need to be modified to better fit the context of older persons’ daily lives, for example. We changed ‘goals’ to ‘wishes’ and excluded ‘needs.’ The difficulty of distinguishing between needs and wishes is also evident in the Bravell et al. (2021) study. Furthermore, it became easier to reflect on what was good or bad in HC and what needs to change in practice to align with individuals’ wishes. The loving struggle during the journey of the knowledge process can create partnerships, bring alternative interpretations, and provide deeper knowledge and insights (Ricœur, 1993), which in turn can lead to a productive flow in the knowledge process.

#### Different ways of learning provide flow and a bottom-up perspective in co-creation of knowledge

The fifth lesson we learned during the journey of discovery was the importance of collecting data in different ways and over an extended time in partnership, with each method enriching the others and creating a flow. This allows for a bottom-up perspective that captures differences between what is said and what is done, leading to deeper knowledge of the situation (see Figure 2). Data collection and reflection took place in cycles of action and reflection (Arnold, 2020; Freire, 1996; Petersson & Rämgård, 2023). This allowed identification of new understandings and solutions to problems (Park, 1999), enabling the development of a new way of working. In this, we were able to follow the process using a diary to track what worked well during data collection and how to interpret what was going on (Polit & Beck, 2017 [2018]), which can be seen as a strength.

The interviews can be likened to genuine dialogues with no predetermined outcomes (Gadamer et al., 2004). Both RFs and co-researchers should be active and learn from each other as they study opportunities and obstacles, what needs to change, and develop knowledge collaboratively (Lindhult, 2022) in a partnership. Participant observations can be described as shadowing staff in their physical environment, observing what they do at work (Czarniawska, 2014; Quinlan, 2008). At the same time, informal conversations were held that can be likened to go-along conversations, about how staff could simultaneously experience and interpret on-going situations (Coker et al., 2017). The informal conversations created flow and brought RFs closer to the staff (Swain & King, 2022). This is a flexible data collection method that is used in the moment; whether cycling, walking, or driving, where ideas could be created and reflected on with staff (Swain & King, 2022). This made it easier to come up with ideas and suggestions of new ways of working according to individuals’ wishes.

The focus group discussions were of great importance in bringing together and refining the data into manageable preliminary themes that highlight opportunities and obstacles. Co-researchers were also invited to join reference groups where they discussed and shared learning with RFs (Heron & Reason, 2008) to clarify knowledge. In the appreciative inquiry circles, the implementation of the new way of working in HC was initiated. There was a risk of a top-down perspective being adopted when first-line managers were involved, as their voices carried the most weight. Nevertheless, persons with the mandate to make decisions need to be involved in the appreciative inquiry circles to facilitate implementation at all levels (Møgster et al., 2024). First-line managers can also offer support with their leadership as structural changes can require a culture shift (Backman, 2018; James et al., 2024b; Wolmesjö et al., 2024). Furthermore, it is a strength that the same co-researchers and RFs participated in several data collection methods as their relationships could develop, allowing experience and knowledge to build over time at both individual and HC levels.

One of the most important lessons learnt, and a major weakness of the study design from the beginning, is that the voices of the older persons and relatives were not heard in focus group discussions and appreciative inquiry circles, which was also pointed out by the staff. This means that the bottom-up perspective and democratic approach were lost. Furthermore, we conducted an analysis inspired by Braun and Clarke’s (Braun & Clarke, 2006) thematic analysis where data was mainly analysed, critically reviewed, and confirmed by co-researchers. Braun and Clarke (Braun & Clarke, 2006) argue that it is a useful method for participatory research, with a large amount of data used to find similarities and differences. Using several different data collection methods allowed for triangulation to create a picture of a complex phenomenon (Polit & Beck, 2017 [2018]). We have met co-researchers from different HC units over a long period of time, which makes the results credible. When the same phenomenon has been studied several times at different HC units, it involves time and space triangulation (Polit & Beck, 2017 [2018]). Member checking has been used as data was compiled continuously. The RFs went back to co-researchers using different data collection methods to avoid losing content and to ensure that co-researchers recognised themselves and that it mattered to them, which enhanced credibility (Stringer & Genat, 2004). When new HC units were included, the data was built on and brought further confirmation of the preliminary result.

#### Having a shared goal in the loving struggle provides flow and enables engagement

The sixth lesson we learned is that anything is possible with a shared goal, and we discovered that research can be conducted remotely. When the pandemic struck, we had to decide whether to cancel or continue the project. Extensive planning and several ethical considerations were made. Lupton (Lupton, 2021) describes ethical difficulties in engaging co-researchers when the organisation is under great pressure and in uncertain times when normal routines do not exist. These situations require more from the co-researchers (Pacheco & Zaimagaoglu, 2020), such as higher levels of emotional engagement (Svenaeus, 2009).

When data collection needed to be re-designed, we had the advantage of being in the field with the co-researchers. Pre-existing relationships with a shared goal and engagement facilitated the transition to digital alternatives. However, it can be more difficult to achieve symmetry and relationships when physical meetings are not possible (Lazarte et al., 2020). The experience is that symmetry was apparent in the telephone interviews that flourished. The co-researchers’ engagement in the research could also be a way to break some of the negative effects of COVID-19 (Hall et al., 2021) and the older persons’ social isolation brought about by the pandemic (Lupton, 2021).

#### Loving struggle is a way to adapt PAAR to changes in the field for practice to evolve

The seventh lesson highlighted that PAAR needs to change and adapt to the field’s context, for practice to evolve. For example, meetings needed to be changed frequently, new RFs were appointed and removed, questions were rephrased, and the project moved to digital alternatives, all during a loving struggle. PAAR involves RFs and co-researchers working together where practice is changed, and implementation takes place over time (Reason & Bradbury, 2008). Successful implementation depends on how well it is anchored among all actors throughout the environment (Ghaye, 2010; Havens et al., 2006; Lindhult, 2022). This is consistent with experiences from previous projects (James et al., 2015; James et al., 2019) where there have been difficulties with implementation as not all HC units in the current elderly care participated. On this basis, we decided to include all HC units in this project. However, including everyone may be impossible, depending on various circumstances. It may be appropriate to start with those who are most interested (Lindhult, 2022). In retrospect, we realise that the idea to have everyone participate in all parts was too ambitious.

**Figure 4.**
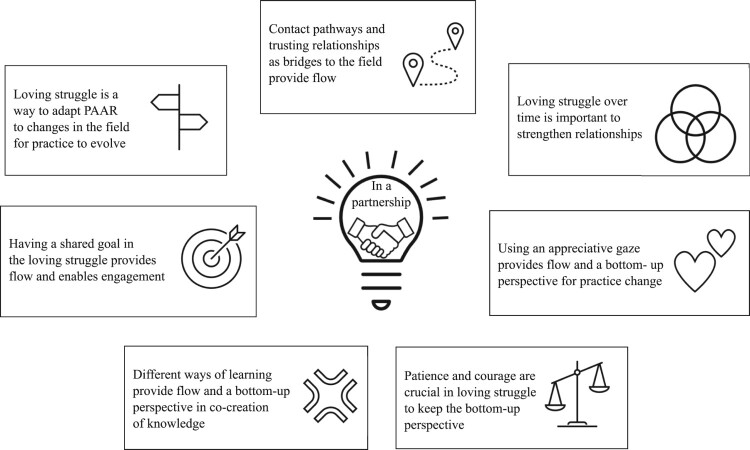
The seven lessons learned in partnership describe what are needed for a flourishing knowledge process in practice.

Finally, the seven lessons learned (see Figure 4) can be context-specific since elderly care is organised differently in different countries. However, at an overall level, the seven lessons can be transferable to other contexts: the need for contact pathways, trusting relationships, appreciative gaze, patience and courage, learning together, having a common goal and engaging in a loving struggle. Independently of the context, the overview of the PAAR process (see Figure 3) can be used as a guide to start projects and to create new ways of working in other contexts for practice to evolve. Despite this, PAAR is an ongoing process in partnership that changes with the context.

## Conclusion

Throughout the journey in partnership, the diary revealed that a tension between a loving struggle and a flow is what drives the cycles of actions and reflections, which are needed for a flourishing knowledge process in practice. The journey of discovery in the PAAR process led to seven key lessons. First, to get flow into the process, RFs need to use contact pathways and build trusting relationships as bridges to the field. During the knowledge process loving struggle arises over time which strengthen relationships. We also learned that an appreciative gaze is a prerequisite for learning together and creating engagement and flow for practice change. In addition, patience and courage are crucial in loving struggle to create the conditions required to keep the bottom-up perspective. RFs and co-researchers need to learn together in different ways, to discover new insights and thereby keeping the bottom-up perspective strengthen the flow. Furthermore, we learnt that engagement is facilitated by a shared goal in the PAAR process. Finally, in practice, everything can change in the field, but through a loving struggle for the shared goal, it is possible to adapt the PAAR process and continue to co-create new knowledge and evolve practice.

## Data Availability

Due to the nature of the research and ethical considerations, no data are available.
